# Late Eocene onset of the Proto-Antarctic Circumpolar Current

**DOI:** 10.1038/s41598-019-46253-1

**Published:** 2019-07-12

**Authors:** Sudipta Sarkar, Chandranath Basak, Martin Frank, Christian Berndt, Mads Huuse, Shray Badhani, Joerg Bialas

**Affiliations:** 1 Department of Earth and Climate Science, Indian Institute of Science Education and Research Pune, Pune, 411008 India; 2 Department of Geological Sciences, California State University, Bakersfield, CA 93311 USA; 3 GEOMAR Helmholtz Centre for Ocean Research, Kiel, 24148 Germany; 4 School of Earth and Environmental Sciences, University of Manchester, Manchester, M13 9PL UK; 5 IFREMER, Unité de Recherche Géosciences Marines, Centre de Bretagne, 1625 Route de Sainte-Anne, 29280 Plouzané, France

**Keywords:** Palaeoceanography, Geochemistry, Stratigraphy, Geophysics, Sedimentology

## Abstract

The formation of the Antarctic Circumpolar Current (ACC) is critical for the evolution of the global climate, but the timing of its onset is not well constrained. Here, we present new seismic evidence of widespread Late Eocene to Oligocene marine diagenetic chert in sedimentary drift deposits east of New Zealand indicating prolonged periods of blooms of siliceous microorganisms starting ~36 million years ago (Ma). These major blooms reflect the initiation of the arrival and upwelling of northern-sourced, nutrient-rich deep equatorial Pacific waters at the high latitudes of the South Pacific. We show that this change in circulation was linked to the initiation of a proto-ACC, which occurred ~6 Ma earlier than the currently estimated onset of the ACC at 30 Ma. We propose that the associated increased primary productivity and carbon burial facilitated atmospheric carbon dioxide reduction contributing to the expansion of Antarctic Ice Sheet at the Eocene-Oligocene Transition.

## Introduction

**Figure 1:**
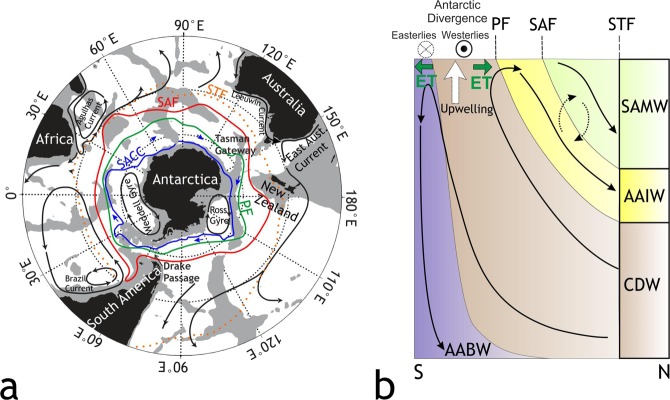
Southern Ocean circulation and circumpolar oceanic fronts. (**a**) The Antarctic Circumpolar Current (ACC) marked by blue arrows and main oceanic fronts from north to south are represented by the sub-Tropical Front (STF), Subantarctic Front (SAF), the Polar Front (PF), and the Southern ACC Front (SACC). Figure adapted from ref.^71^. (**b**) Simplified schematic representation of the present-day Southern Ocean overturning circulation^3,5^. Ekman transport (ET) resulting from the Antarctic Coastal Current flowing counter-clockwise around Antarctica under the influence of the Polar easterlies causes waters to move towards Antarctica, while a northbound Ekman transport resulting from an eastbound ACC drives the waters away from Antarctica. It creates an area of divergence called the Antarctic Divergence Zone, where the Circumpolar Deep Water (CDW) upwells to the surface south of the Polar Front (PF). Northward advection of nutrient-rich upwelled water takes place by Ekman transport^71^ and references therein. Its subsequent subduction to intermediate depths forms the Antarctic Intermediate Water (AAIW). Part of the upwelled waters moves southwards forming the Antarctic Bottom Water (AABW). During the northward advection, some part of the Antarctic surface water mixes with subtropical surface water to form Subantarctic Mode Water (SAMW).

Today, the westerlies-driven Antarctic Circumpolar Current (ACC) is the volumetrically largest geostrophic current, which connects the Atlantic, Indian and Pacific Oceans (Fig. 1a). It plays an important role in the global distribution of heat^1^, nutrients, salt, carbon, as well as in the gas exchange between the atmosphere and the ocean and has thus exerted a strong influence on Earth’s climate^2,3^. The isopycnal tilt of water masses within the ACC prevents substantial subtropical surface heat from reaching Antarctica facilitating its thermal isolation, cooling, and stabilization of the Antarctic glaciers^4^. The complex interaction between the westerlies, the ACC and the bottom topography supports upwelling of nutrient-rich Circumpolar Deep Water at the Antarctic Divergence Zone (Fig. 1b), making it one of the biologically most productive regions of the world ocean^5,6^. The ongoing rapid climate change^7^ may strengthen the southern hemisphere westerlies^8,9^, which would influence the strength of upwelling, heat and carbon transport, and diminish the stability of the ice shelves around Antarctica^10^. Although the ACC has been a key component of present-day and past ocean circulation and climate, the timing of its onset and progressive development are poorly constrained. Moreover, the accurate reconstruction of the onset of the ACC is fundamental for our understanding of the long-term paleoceanographic evolution and climatic repercussions including major cooling during the Middle and Late Eocene^11^ and the development of continental-scale Antarctic glaciation^12^.

The initiation of the ACC depended on the tectonic opening of the Drake Passage and Tasmanian Gateway (Fig. 1a). The onset age of the ACC is highly debated, and there is a wide range of estimates between 41 Ma refs^13,14^ and 23 Ma ref.^15^ due to an incomplete understanding of basin evolution and paleoceanography. A fully-developed modern ACC likely prevailed shortly after 30 Ma when the deep open Tasmanian Gateway aligned with the westerlies^16^. However, the development history of an eastbound proto-ACC, an intermediate-depth precursor of the modern configuration^16^ is unclear. Earliest estimates place it between 41 and 37 Ma refs^14,17–19^ that coincides with deepening of the Drake Passage to intermediate depths. Major subsidence of the Tasmanian Gateway at 35.5 Ma refs^20,21^ probably facilitated proto-ACC development. However, the eastbound Tasmanian throughflow likely to have initiated at 30 Ma ref.^16^, thereby, casting doubt on the existence of a proto-ACC. Here, we present reflection seismic analyses of marine sedimentary successions in the southwestern Pacific sector of the Southern Ocean to test the hypothesis that a proto-ACC existed during the Late Eocene and Early Oligocene (35-30 Ma), thus preceding the modern ACC^16,22^. We present evidence for spatial and temporal variations of paleo-ocean circulation and marine bio-productivity related to the establishment of the proto-ACC, which was driven by the opening of the Tasmanian Gateway and had consequences for the expansion of the Antarctic Ice Sheet during the Late Eocene-Early Oligocene.

## Results

**Figure 2:**
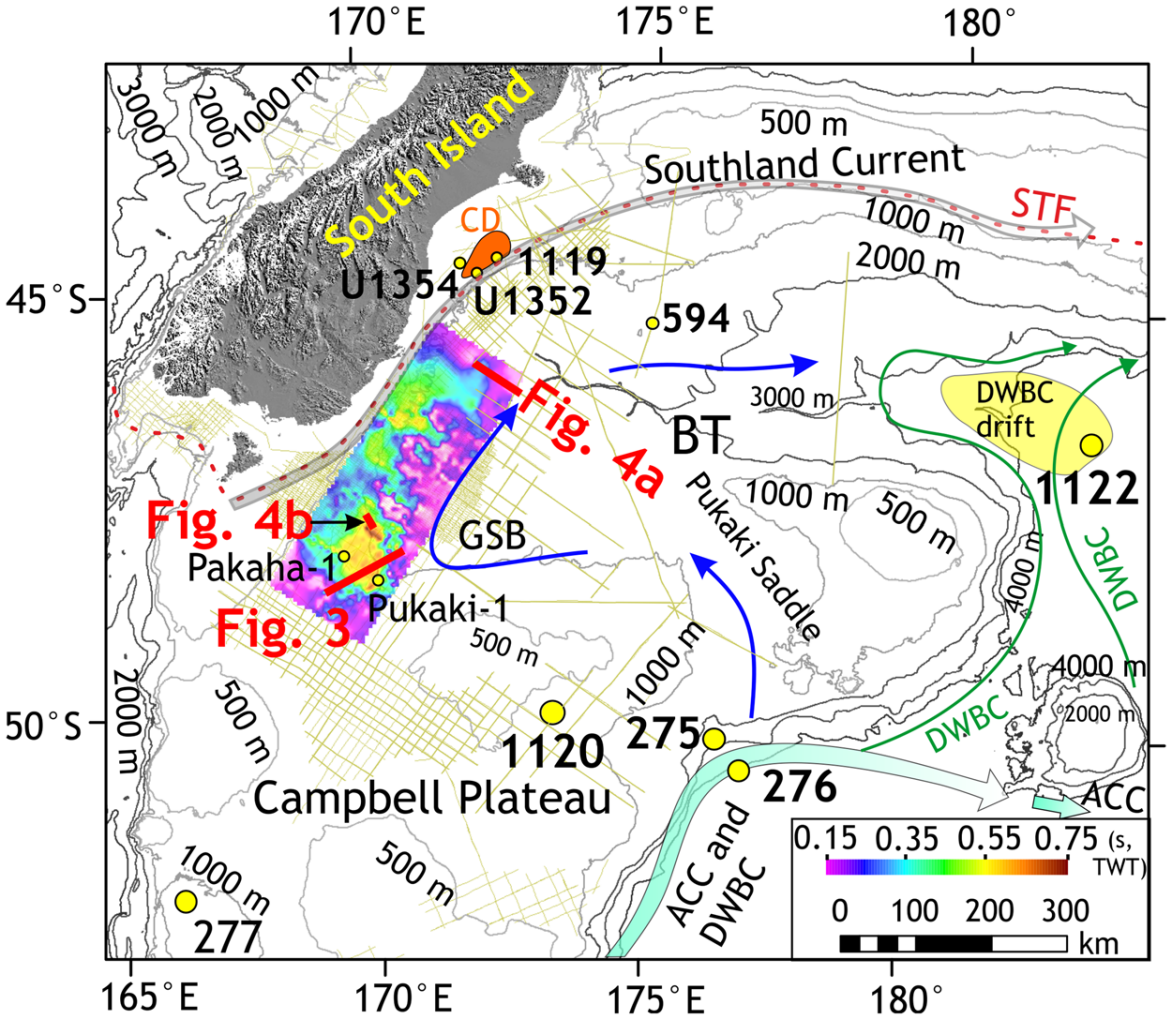
Study area map. Map showing the South Island of New Zealand, Campbell Plateau, sub-Tropical Front (STF)^72^, Antarctic Circumpolar Current (ACC), Pacific Deep Western Boundary Current (DWBC)^73,74^ and a modern cyclonic circulation (marked by blue arrows) in the Great South Basin (GSB) and the Bounty Trough (BT). Canterbury drifts (CD)^59^ and DWBC drifts^28^ are shown. Time thickness (two-way travel time in seconds) map of the mid-Eocene to Late Eocene interval in the GSB shows the NNE–SSW striking elongate sedimentary drift. Subsidiary drifts were deposited on the eastern offshore side of the main mound. Boreholes of ODP Leg 181 (1119, 1120 and 1122), IODP expedition 317 (U1352 and U1354), DSDP Leg 29 (275 and 276), Leg 90 (594) and oil exploration wells (Pukaki-1 and Pakaha-1) are marked.

**Figure 3:**
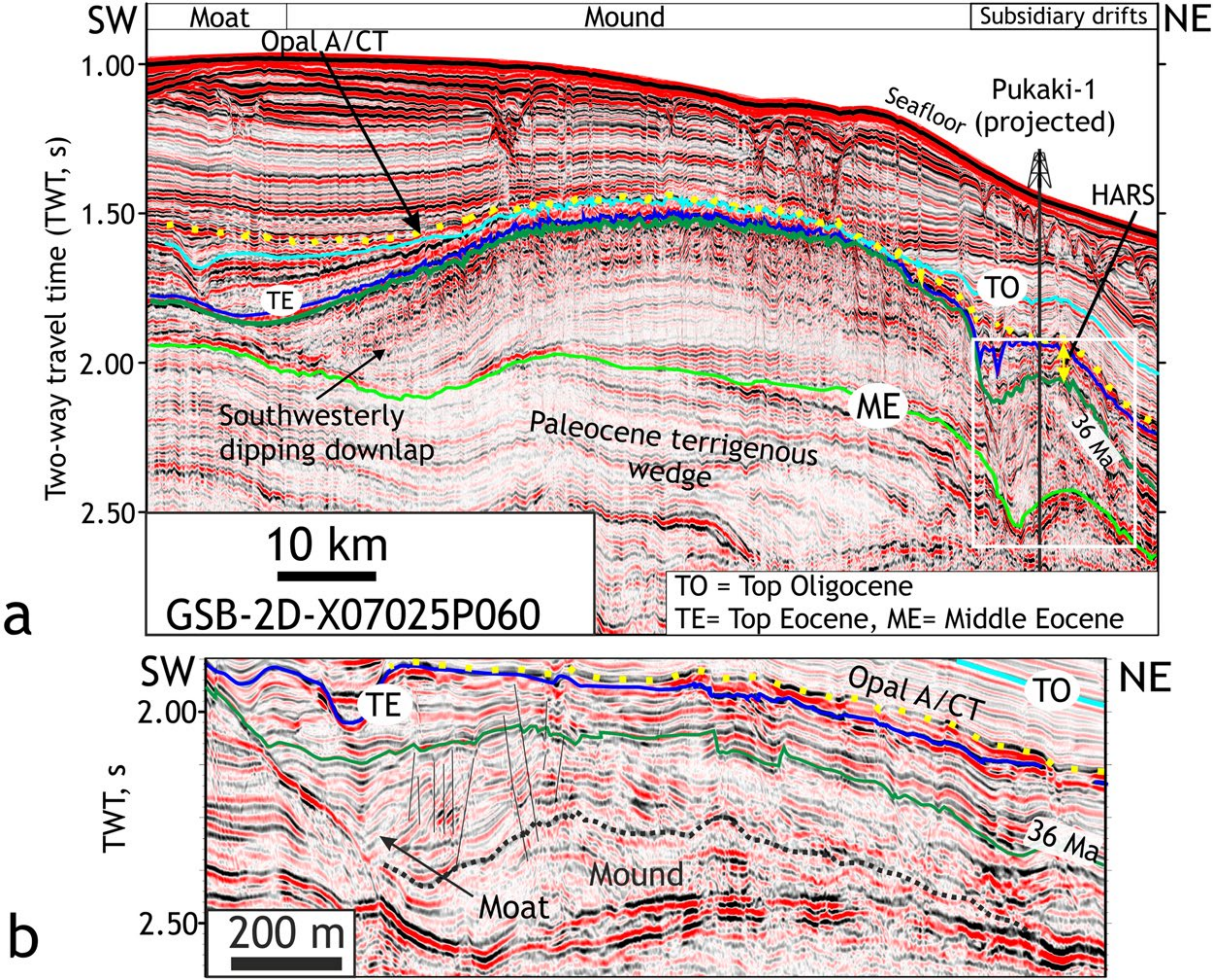
Seismic characteristics of late Paleogene sedimentary drifts. (**a**) A seismic line shows a central mound between markers ME and TE with internal upslope prograding configuration. High amplitude reflections (HARS) occur towards the top of the mound. A moderate to strong positive amplitude reflection is identified as the opal-A to opal-CT reaction front (yellow dotted horizon). We correlated seismic horizons in the drift to Pukaki-1 (Fig. 5a). (**b**) Subsidiary mound developed offshore of the central mound (Location marked by a box in Fig. 3a). It shows internal convex reflection pattern and a landward moat. High amplitude reflections are seen above the marker that is assigned an age of 36 Ma.

**Figure 4:**
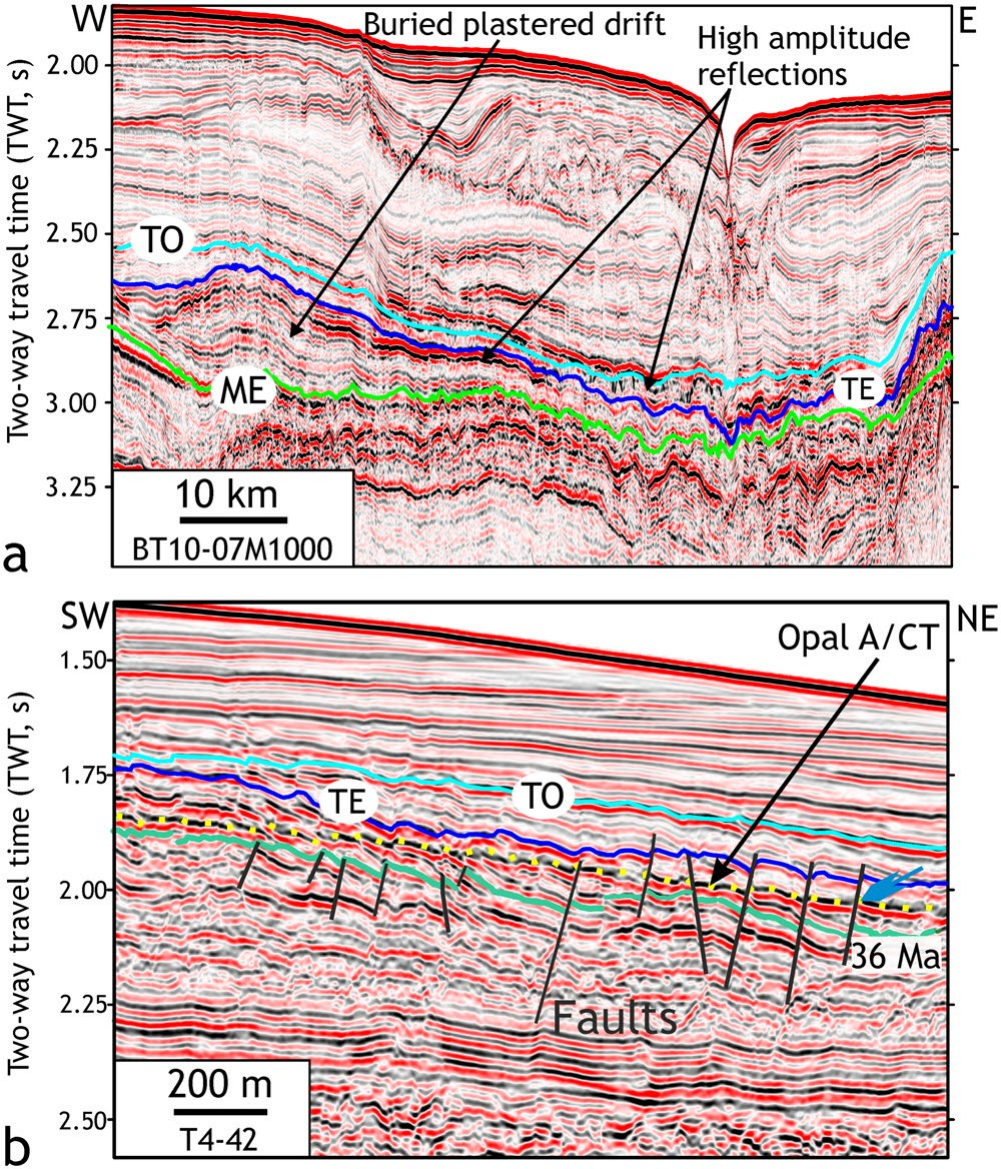
Late Paleogene sedimentary drifts and opal-A/opal-CT reflector. (**a**) A lenticular shaped unit between markers ME and TE is identified as a buried plastered drift south of the Bounty Trough. Strong positive amplitude reflections are seen towards the top of the drift in the BT. (**b**) The opal-A/opal-CT reaction front shows negligible or smaller offset (blue arrow) than the offset of the host Late Eocene unit (cf. ref.^75^). The diagenetic transformation could have post-dated the displacement across the faults, or the displacement rate of the faults was greater than the upward advancement of the diagenetic front. Some faults extending into the Oligocene and Miocene sequence also affected the diagenetic front, probably post-dating diagenetic transformation.

**Figure 5:**
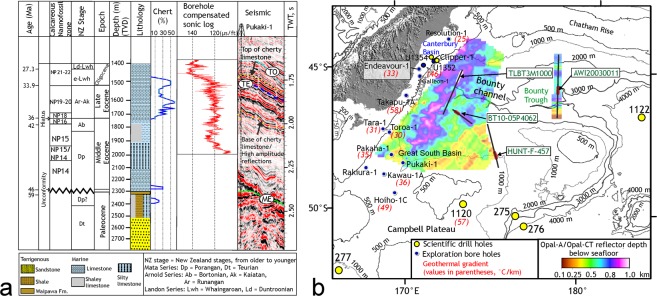
Results from the borehole Pukaki-1 and the spatial extent of an opal-A/opal-CT reflector. (**a**) On the seismic panel, bright reflections within Late Eocene and Early Oligocene strata are correlated with diagenetic chert as determined from recovered rock samples (ages calibrated to ref.^60^). The top of cherty limestone clearly defines the opal-A/opal-CT conversion boundary and correlates with a sharp drop in sonic log response. (**b**) Variation of the depth of the opal-A/opal-CT reflector below the seabed.

Two-dimensional seismic lines (number = 111) (Materials and methods) over the Great South Basin (GSB) and the Bounty Trough (BT) show an elongate, slope-parallel sedimentary body at present water depths of 1–2 km that developed in deep basins east of New Zealand’s South Island (Figs 2, 3a). The internal structure of the sedimentary wedge can be laterally subdivided into three zones: a landward moat, a central mound, and subsidiary mounds in the deeper basin (Fig. 3a,b). The central sedimentary body shows asymmetric external mounded (Fig. 3a) and lenticular shape (Fig. 4a). The subsidiary mounds show a basal zone comprising small (<400 m thick, ~15 km wide) asymmetric mounded external shape and channel-like moats on their landward sides, which developed close to the toe of a Paleocene terrigenous wedge (Fig. 3b). A summary of biostratigraphic and lithologic results of the sedimentary body obtained from the petroleum industry borehole Pukaki-1 well is shown in Fig. 5a (additional details of the sedimentary drift system are provided in Supplementary Text 1). The base of the sedimentary wedge in the central GSB is marked by an erosional unconformity and a prolonged hiatus (59-46 Ma) in the Pukaki-1 well. Internal upslope prograding and downlapping reflections (Fig. 3a) indicate a contourite drift system was deposited by along-slope flowing bottom currents during the Middle-Eocene. From the shape, the orientation of the drift bodies, and their location on the western margin of the basin, we infer that the bottom currents were flowing in a northeasterly, contour-parallel direction with the drift deposit growing on its left due to Coriolis forcing. In the central GSB, the flow turned from northwest to north and then to northeast forming a loop that decelerated the current and resulted in sediment deposition at the outer rim of the loop. The loop is analogous to the modern cyclonic circulation within the GSB and BT (Fig. 2).

A prominent zone of bright reflections between Late Eocene and Oligocene strata (36-30 Ma) within the drift represents chert that overlies shaley limestones devoid of chert at the Pukaki-1 well (Figs 3a,b and 5a). A positive polarity reflection at the top of the chert can be traced regionally and shows both discordant (such as cross-cutting reflection, Figs S1a,b,d and 4b), and concordant (Fig. S1c) relationships with the host strata. The reflection is a typical example of opal-A (biogenic silica) to Cristobalite and Tridymite (opal-CT) diagenetic transformation boundary^23^, which is widespread in the deep waters of the GSB and BT (Fig. 5b). The transformation involves the dissolution of biogenic opal-A, and the precipitation of the microcrystalline, pore-filling opal-CT^24,25^. The transformation causes a reduction in porosity, an increase in sediment bulk density and seismic velocity resulting in an increase in acoustic impedance (product of density and seismic velocity) that causes a high amplitude reflection (Supplementary Text 2). A discordant reflector, cross-cutting stratal reflections indicates post-depositional diagenetic changes rather than inorganic precipitation of chert. The base of the diagenetic transformation zone in the GSB (Fig. 5a) is marked by a substantial reduction in reflection amplitude that correlates with decreasing chert concentration indicating the absence of initially deposited siliceous microfossils within the late Middle Eocene sequence. Using regional seismic lines, we were able to trace the diagenetic transformation zone of silica from the GSB to the Canterbury Basin (CB). The occurrence of Late Eocene and Oligocene chert is a consequence of basin-wide diagenetic transformation (Supplementary Text 3) of originally deposited abundant biogenic opal, indicating a remarkable and prolonged episode of biogenic silica productivity.

**Figure 6:**
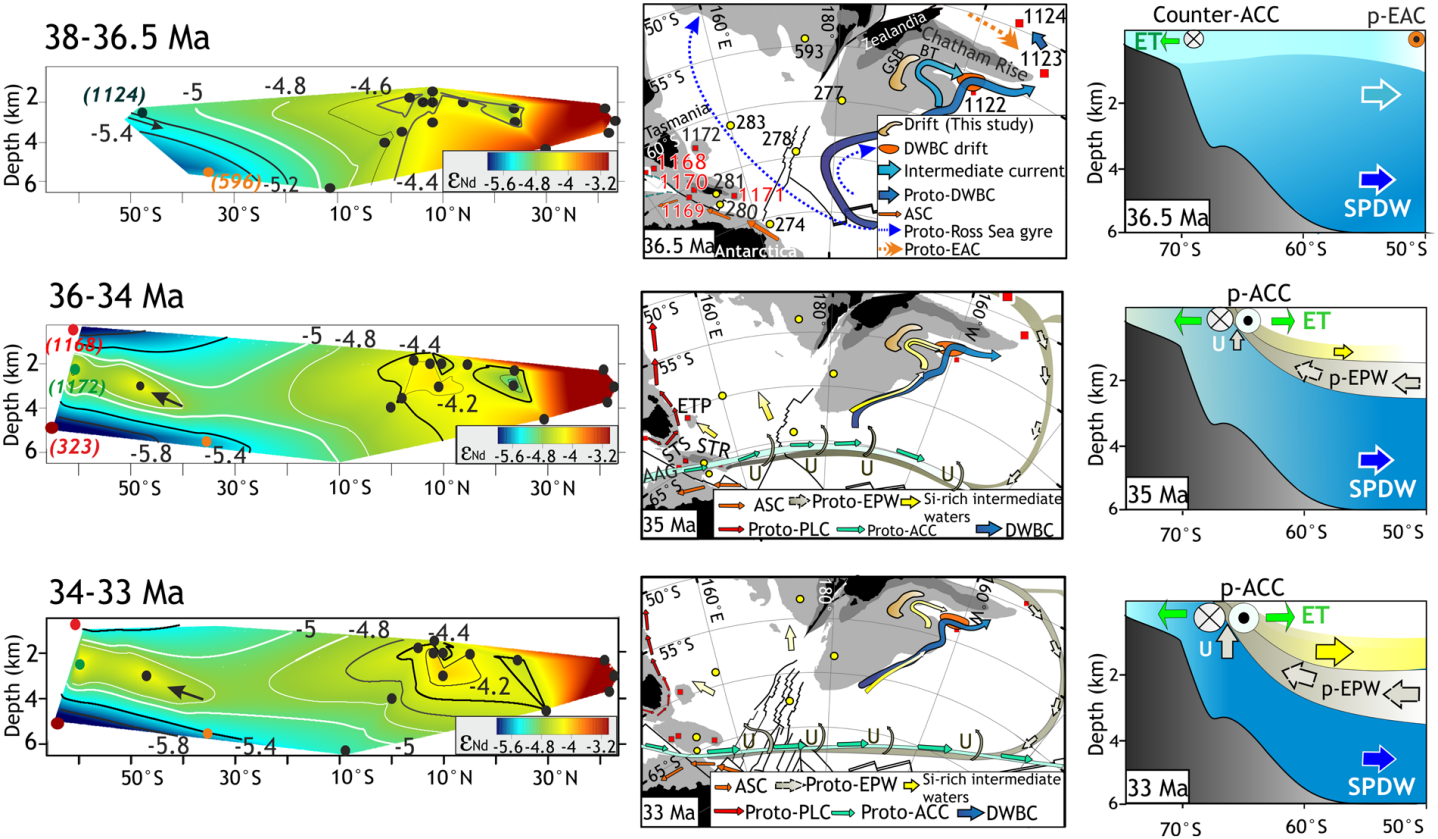
Neodymium isotopic sections for different geologic time bins interpolated using natural neighbor interpolation (Table S1) and schematic late Paleogene southwest Pacific circulation showing the progressive development of a proto-ACC. **(Top)** The 38-36.5 Ma Nd isotopic section (left) reveals the extent and mixing of North and South Pacific deep waters (Fig. S2b)^16,26^. At Site 1124 northbound flow of less radiogenic South Pacific deep waters is indicated by an arrow. Contourites in the GSB and BT are deposited by northbound bottom currents (centre). A westbound Antarctic Slope Current (ASC) existed north of Antarctica. At Site 1124, surface currents were influenced by the proto-East Australian Current (EAC)^36^; while deep northbound currents transported southern sourced deep waters. Cross-section (right) shows the northbound flow of South Pacific deep water (SPDW). **(Middle)** Equatorial/sub-equatorial deep waters with more radiogenic Nd isotopic signature arrive south of 30° S (36-34 Ma) and indicated by an arrow. A proto-ACC started to develop across the STR (centre) causing entrainment and upwelling (U) of proto-equatorial Pacific deep water (Proto-EPW) and subsequent northbound Ekman transport (right). The submerged Chatham Rise deflected the proto-EPW towards east. **(Bottom)** The 34-33 Ma Nd isotopic section (left) is similar to the 36-34 Ma section. A stronger proto-ACC caused upwelling (centre) and pronounced entrainment of proto-EPW (right). Colour codes for the paleogeographic maps^76^ in the middle column: black = land, dark grey = shelf, light grey = slope or submarine rise, white = deep ocean.

The dissolved neodymium (Nd) isotopic composition of open ocean deep waters behaves conservatively, and Nd isotopes have thus been widely applied as tracers of present and past water mass mixing (see Methods for details). Here, we use published Paleocene–Early Oligocene seawater Nd isotope records obtained from fossil fish teeth/bones (henceforth ‘fish debris’) to reconstruct the presence and mixture of southern sourced vs. northern sourced Pacific water masses (Fig. S2a,b). Previously published fossil fish debris Nd isotope signatures (^143^Nd/^144^Nd, expressed as ε_Nd_(t)) for the Paleocene–Eocene (Fig. S2a,b) show that the southern Pacific was bathed by less radiogenic (ε_Nd_(t) = −6.5 to −5.0) deep waters of southern origin while northern Pacific deep waters were more radiogenic (ε_Nd_(t) = −3 to −4.0) refs^26,27^. The ε_Nd_ range of Equatorial Pacific Water is intermediate between these two end members. During the Late Paleocene, the equator-bound proto-Deep Western Boundary Current flowed over the eastern flank of Zealandia^28^ and the Hikurangi Plateau^29^ and transported southern sourced proto-Ross Sea bottom waters^26^. The available Nd isotope data from ODP Sites 1172 between 52-47 Ma (ε_Nd_ = −5.3 to −7) ref.^30^ and from the Hikurangi Plateau (ODP Site 1124) at 36.5 Ma (ε_Nd_ = −5.13 ref.^16^) clearly show the influence of South Pacific deep waters (Figs S2a,b, S3 and 6). The southern sourced less radiogenic waters flowed northward and mixed with radiogenic deep waters in the Equatorial and North Pacific regions. However, more radiogenic signatures (ε_Nd_ = −4 to −4.5) at southern Pacific Site 1124 (water depth = ~3 km) between 36 and 30 Ma are more akin to northern sourced Equatorial Pacific waters^31^ (Fig. S2b). The continuous presence of more radiogenic Equatorial Pacific waters between 36 and 30 Ma is prominent in the Nd isotope records of ODP sites 1124 and 1172 ref.^16^ from the southwest Pacific (Fig. 6).

### Earliest evidence of intermediate-depth circulation in the southwest Pacific

Proto-Deep Western Boundary Current had been active since 65 Ma driving drift deposition at greater depths (>2 km) ref.^28^, however, it was not until the mid-Eocene (~45 Ma) that the first evidence of a persistent southern Pacific intermediate-depth circulation became prominent. We argue that the onset of contourite deposition in the GSB and BT was a result of cyclonic flow path initiated by circulation changes at intermediate depth. Enhanced basin subsidence (~1 km) between 55 and 45 Ma refs^32,33^ lead southern-sourced bottom-currents to be channelized into the GSB across the Pukaki Saddle (Fig. 2) and circulated along a clockwise path (Supplementary Text 1). Evidence of a cyclonic circulation within the BT (Fig. 2) emerging from the northern edge of a fully-developed ACC has only been reported since the Early Miocene^34^. Our finding of a similar cyclonic flow path in the same region thus pre-dates the earlier known cyclonic circulation^35^ by as much as 20 million years.

In order to explain the origin of mid-Eocene and Oligocene (45-30 Ma) drift deposits (Fig. 3), we invoke a persistent northbound intermediate-depth flow between the topographically constrained deep boundary current^28^ and the proto-Ross Sea gyre^34,36^. The Pacific ε_Nd(t)_ compilation for 45–47 Ma (Fig. S3) suggests that deep-water masses with distinct Nd isotopic signatures (Fig. S2a) formed in the high South and North Pacific latitudes, representing a situation similar to the Early Eocene (~53 Ma) ref.^26^. The proto-Deep Western Boundary Current transported the southern-sourced waters further north and a branch of that current circulating the Campbell Plateau was able to enter the GSB during a period of anomalous basin subsidence (55-45 Ma, Supplementary Text 1). The onset of deposition of the southwest Pacific drifts in the GSB at ~45 Ma provides strong evidence for an active equatorward flow of Southern Pacific deep and intermediate waters when the Tasmanian Gateway was still closed.

### Critical paleoceanographic changes during Late Eocene to Oligocene

The mid-Eocene–Oligocene section within the drift deposits allows us to evaluate the time of the establishment of the early ACC and consequent overturning circulation that has played a central role for Southern Ocean upwelling. Diagenetic chert is confined to Late Eocene and Early Oligocene strata within the sedimentary drifts of the GSB and BT but is rare in deeper levels, indicating the original scarcity of biogenic opal deposition. During 55-47 Ma silica productivity was mostly restricted in shallow marine continental margins of the Australo-Antarctic Gulf and the southwestern Pacific where nutrients were primarily supplied from local terrigenous sources^37–39^. The open ocean deep silica deposition in South Pacific has been reported from 47-35 Ma. The open marine silica deposition was a result of an increase in ocean-wide surface productivity in the tropical and southern high-latitudes^38,40^. In spite of wide-scale silica deposition in a deep marine setting, chert is still absent in the GSB deposits between 45-36 Ma (Fig. 5a). During this period transport of silicic acid from the tropical/sub-tropical regions to the surface waters of the southern high-latitudes was controlled by the proto-Ross gyre^36^. Bio-utilization of silica during long transport along the proto-Ross gyre limited the amount of silicic acid eventually reaching the surface waters of the GSB and BT. The prominent transition from calcareous to siliceous limestone in the Pukaki-1 well in the GSB occurred after ~36 Ma indicating an increase in the nutrient content of the surface ocean at this location. Silica-rich sub-equatorial waters must have bypassed the proto-Ross gyre and probably used a more efficient upwelling pathway to arrive at the high-latitude South Pacific (Supplementary Text 3).

Support in favour of this observation and probable mechanism comes from meridional composite sections of ε_Nd(t)_ records^16,26^ (Fig. 6). At ODP site 1124, relatively less radiogenic waters (ε_Nd(t)_ = −5.13 at 36.5 Ma) at ~2.5 km water depth (Supplementary Table S1) were replaced by an enhanced incursion of more radiogenic sub-equatorial Pacific deep waters (ε_Nd_(t) = −4 to −4.5) between 36 and 33 Ma (Fig. 6). During 36-30 Ma more radiogenic waters circulated in the high-latitude South Pacific Site 1172 compared to the waters during early Middle Eocene (Fig. S2) refs^16,30^. While these radiogenic values in high-latitude southwest Pacific can be due to terrigenous input from east Antarctic terrains^41^, the candidate bedrocks are of lower Paleozoic origin with extreme negative Nd isotopic signatures (ɛ_Nd_ = −11.2 to −19.8), hence do not support the observation. The McMurdo volcanics (ɛ_Nd_ > 0) is another potential source capable of altering the deep water signal in the southwest Pacific; however, volcanic activity and dispersal of volcanic materials are not reported around the Ross Sea until Oligocene/Miocene (~24 Ma) ref.^42^. Presence of Ferrar Group sediments (ɛ_Nd_ = −3.5 to −6.9) is only known in the Cape Roberts cores in the Ross Sea since Early Oligocene (~31 Ma) ref.^43^. Thus, all probable terrigenous sources are either not radiogenic enough or their presence in the Ross sector postdates the observed Late Eocene Nd isotopic change to radiogenic values in the southwest Pacific. Therefore, the Late Eocene presence of a more radiogenic water mass at ~2.5–3 km at Sites 1124 and 1172 indicates advection of waters from a northern source. This contention is also supported by the contemporaneous occurrence of temperate to sub-tropical diatom species, and absence of sub-Antarctic/Antarctic foraminifera at Site 1124 ref.^44^.

Thus, it is fair to say that the mechanism that led up to enhanced biosiliceous deposition in the GSB at ~35 Ma was controlled by upwelling of sub-equatorial Pacific deep waters in the high-latitude South Pacific (Proto-EPW, Fig. 6). The deep, old sub-equatorial North Pacific waters were enriched in silicic-acid and nutrients^45^, which are reflected in light benthic foraminiferal δ^13^C values^46^. The proto-EPW outcropped near high-latitude southwestern Pacific, which would require an emerging proto-ACC and the combined effect of isopycnal tilt and Ekman driven pull (Fig. 6). Part of the upwelled waters was transported northward due to Ekman divergence. Local cooling around Antarctica linked to early ephemeral ice sheets^11,47^ likely facilitated surface ocean cooling and sinking of those upwelled waters to intermediate depths towards the East Tasman Plateau (Site 1172) and the deep basins east of New Zealand’s South Island. The sinking of the proto-EPW to intermediate depths at the East Tasman Plateau is also indicated by improved ventilation at site 1172 after 36 Ma ref.^20^. Further evidence of silicic acid-rich water masses came from widespread diatom proliferation and enhanced biogenic opal deposition in the contemporaneous deep-marine sedimentary record east of New Zealand after 36 Ma (Figs 5b and 6).

### Tasmanian throughflow and onset of proto-ACC

The onset of the proto-ACC requires (i) submergence of the land bridge between the South Tasman Rise (STR) and Antarctica, (ii) westerlies-driven eastbound Tasmanian through-flow, and (iii) deepening of the Tasmanian Gateway to intermediate depth. A shallow marine connection between the Australo-Antarctic Gulf and the southwest Pacific across the southwestern STR existed since ~38 Ma refs^21,48^. Evidence of contourite deposition (~38 Ma) in the southwestern STR indicates bottom-currents flowing from the Australo-Antarctic Gulf to the southwest Pacific (Fig. S4, Supplementary Text 4). Under a globally warm Eocene climate, the zone of the westerly winds likely lay close to the Polar high-latitudes (approximately 60–65°S refs^39,49,50^), and would have influenced much of southern Australia, southwestern STR and facilitated the eastbound Tasmanian throughflow (Fig. 6). The major tectonic deepening of the Tasmanian Gateway together with strong westerlies at ~35.5 Ma ref.^21^ had set the boundary conditions for the development of a proto-ACC. Modeling results show that advection of deep subtropical waters into the southern Pacific via an intermediate-depth proto-ACC was possible^51^. An already open Drake Passage^14^ likely provided strong zonal support to the newly-established eastbound Tasmanian throughflow.

Our results indicate a major upwelling of silica-rich sub-equatorial water at the high-latitude South Pacific was related to the development of a proto-ACC, which pre-dates the modern ACC by ~5–6 Ma^16^. Report of the Late Eocene ‘opal pulse’ at ODP Site 1090 in the southern Atlantic was attributed to enhanced productivity and opaline silica accumulation due to a southbound extension of the proto-Indian Ocean Equatorial waters^52^. The timing of the southern Atlantic opal pulse coincided with the beginning of intermediate depth flow through the Drake Passage (~37 Ma), that is supported by Nd isotope data^13^. The Late Eocene silicon isotope gradient between the Agulhas Ridge (ODP Site 1090) and the Maud Rise (Site 689) has been interpreted as a result of substantial upwelling of silicic acid rich deep waters associated with a nascent ACC and bio-utilization^53^. Evidence supporting the development of a proto-ACC and meridional overturning in the South Atlantic Ocean also emphasizes the role of early circumpolar currents for southern hemisphere circulation starting at ~36 Ma ref.^54^. Thus, the South Atlantic record corroborates our findings in the southwestern Pacific.

The emplacement of a proto-ACC at ~35.5 Ma likely paved the way for oceanographic changes that facilitated the development of the Early Oligocene Antarctic glaciation. The proto-ACC driven upwelling could release CO_2_ from the deep ocean to the atmosphere (cf. ref.^55^). At the same time, however, upwelling related phytoplankton production increased in the high-latitude Pacific region of the Southern Ocean^39^, the sedimentary basins east of New Zealand’s South Island as well as in other sectors of the Southern Ocean^56–58^, making the high-latitude ocean a net sink of atmospheric CO_2_ ref.^53^. If these conditions prevailed long enough, atmospheric CO_2_ drawdown contributed to a global cooling trend culminating in the glaciation and expansion of the Antarctic Ice Sheet ~33.7 million years ago.

## Materials and Methods

### Seismic data and interpretation

We interpreted a regional set of two-dimensional seismic lines and analysed information from petroleum industry boreholes and scientific drilling results in the basins east of New Zealand’s South Island. A regional database containing open file petroleum industry exploration wells, seismic data across New Zealand was obtained from New Zealand Petroleum Exploration (http://www.nzpam.govt.nz/). We added additional seismic lines from the Bounty Trough region^34^ and the Canterbury Basin^59^ to this database in order to aid stratigraphic correlation and interpretation. Seismic data were acquired during the OMV10, OMV8 and DUN6 surveys in the Great South Basin using airgun arrays (total volume ~66 l) with a shot interval of 25 m. The TLBT survey was conducted with airgun arrays (total volume ~88 l) and the shot interval was 37.5 m. The TLBT survey used a 10 km long streamer with 800 hydrophone groups at a spacing of 12.5 m, whereas OMV10, OMV08 and DUN06 surveys were conducted with a 6 km long streamer and 240 hydrophones (group interval 25 m). The record length during the TLBT survey was 12 s (sample rate 2 ms), while record length during OMV10, OMV8 and DUN6 surveys was 8 s (sample rate 2 ms). Data processing comprised resampling from 2 ms to 4 ms, trace quality control, rejection of bad traces, swell noise attenuation in shot and receiver domains, initial gain recovery to compensate for amplitude decay (T squared compensation for inelastic attenuation and spherical divergence losses), Common Mid-Point binning (CMP bin size 12.5 m) and sorting into CMP domain, F-X interpolation in CMP domain, frequency filtering (band pass filter for 0 to 1000 ms TWT with 6–90 Hz and for 1000 to 6000 ms with 4–50 Hz), semblance velocity analysis (1 km interval), radon demultiple, migration velocity analysis (1 km interval), prestack Kirchhoff time migration, residual moveout correction and stack. True amplitudes were preserved during pre-stack time migration.

The regional database contains an integrated mapping of key age based horizons on a regional basis for 77 seismic lines. We performed additional seismic well ties in the Great South Basin, the Canterbury Basin, and the Bounty Trough and ages were assigned with the biostratigraphic records from exploration wells Pukaki-1 and Pakaha-1 in the Great South Basin and two scientific drill sites (Deep Sea Drilling Project site 594, Ocean Drilling Program site 1119). Seismic stratigraphic correlation enabled us to trace regional seismic horizons from the Great South Basin to the scientific boreholes 1119, U1354 and U1352. The biostratigraphic (nannoplankton zones) information obtained from the Pukaki-1 and Pakaha-1 wells were correlated with the information from the borehole U1352 to validate stratigraphic ages, which were calibrated to ref.^60^. Additional seismic lines from the South Tasman Rise south of Tasmania were obtained from the Australian Geological Survey Organisation.

### Neodymium (Nd) isotope data compilation

In the modern ocean, major deep ocean water masses such as North Atlantic Deep Water (NADW, ε_Nd_ = ~ −13.5) and North Pacific deep water (NPDW, ε_Nd_ = −4)^61,62^, and references therein are associated with distinct Nd isotope compositions. Dissolved seawater Nd isotope studies indicate that water masses below the thermocline and within the global overturning circulation pathway closely reflect values predicted from mixing of these end members at varying proportions. Thus, Nd isotopes in open ocean deep waters are a robust water mass tracer despite their potential to be altered by inputs from local sources close to the ocean margins (e.g., boundary exchange)(ref.^63^ and references therein). If seawater Nd isotopes are faithfully archived in sediments, it is possible to apply them for reconstructing past ocean circulation.

Among the many sedimentary archives used to extract pristine Nd isotopic signature of past ocean bottom waters, fossilized fish teeth and bones (i.e., fish remains) are considered most reliable(e.g.,^64,65^). Fossil fish remains are made up of hydroxy-fluorapatite and acquire their Nd signature during early diagenesis while the biogenic-phosphates are still in contact with the bottom water^64^. Thus, the Nd isotope composition of fossil fish remains represents a bottom water signature (Nd isotopes expressed as ε_Nd_(t) = [(^143^Nd/^144^Nd)_sample_/(^143^Nd/^144^Nd)_CHUR(t)_ − 1] * 10^4^), with (^143^Nd/^144^Nd)_CHUR_ = 0.512638 representing the Chondritic Uniform Reservoir (CHUR)^66^ and CHUR(t) represents the age-corrected CHUR value). Hydrogenetic ferromanganese crusts obtain their Nd isotope signature from the ambient seawater during their growth and are also suitable for reconstructing seawater tracer histories^67^.

Here, we compiled published fossil fish teeth Nd isotope data from the Pacific Ocean^26,68,69^) and Nd isotope data from the central Pacific ferromanganese crusts^67,70^) to produce paleo-latitudinal sections for four time bins 47-45 Ma, 38-36.5 Ma, 36-34 Ma, and 33–34 Ma. Three additional sites providing fish teeth Nd isotopic records and age models in the southwest Pacific from Ocean Drilling Program sites 1124 (Hikurangi Plateau), 1168 (Western Tasmanian Margin) and 1172 (East Tasman Plateau) were adopted from ref.^16^ in order to constrain the timing of the intrusion of water masses from the Australo-Antarctic Gulf (AAG) into the South Pacific. The time slice sections are interpolated on a paleo-latitude and depth grid using natural neighbor interpolation. In addition, seismic data were used to determine bottom water flow directions at the Tasmanian Gateway sites to support the interpretations drawn from Nd isotopic compositions.

## Supplementary information

Supplementary Material
