# Supplementary material for: Late Eocene onset of the Proto-Antarctic Circumpolar Current
Source: Sci Rep. 2019 Jul 12;9:10125. doi: 10.1038/s41598-019-46253-1 (PMC6626031; doi:10.1038/s41598-019-46253-1)
Supplement: Supplementary file 1 — Supplementary Material [file 41598_2019_46253_MOESM1_ESM.doc]

**Supplementary material:**

# Late Eocene onset of the Proto-Antarctic Circumpolar Current

Sudipta Sarkar^1*^, Chandranath Basak^2^, Martin Frank^3^, Christian Berndt^3^, Mads Huuse^4^, Shray Badhani^5^, Joerg Bialas^3^

^1^Department of Earth and Climate Science, Indian Institute of Science Education and Research Pune, Pune 411008, India

^2^Department of Geological Sciences, California State University, Bakersfield, CA 93311, USA

^3^GEOMAR Helmholtz Centre for Ocean Research, Kiel 24148, Germany

^4^School of Earth and Environmental Sciences, University of Manchester, Manchester M13 9PL, UK

^5^IFREMER, Unité de Recherche Géosciences Marines, Centre de Bretagne, 1625 Route de Sainte-Anne, 29280 Plouzané, France

*Correspondence to: sudipta@iiserpune.ac.in

**Supplementary Text 1**

**Late Paleogene sedimentary drift system in the basins east of New Zealand’s South Island**

The interpretation of several two-dimensional seismic lines over the Great South Basin (GSB) and the Bounty Trough (BT) allowed identification of the internal structure and external shape of a sedimentary wedge. The central wedge reveals a mounded external shape in the central GSB at present water depths of ~1000 m. The core of the central mound shows gently convex external shape, which is asymmetrical to the southwest and an internal upslope accreting downlap pattern. An elongate, lenticular shaped sedimentary unit shows internal onlap on a south-easterly dipping paleoslope (marker ME) immediately south of the BT region (**Fig. 4a**).

Based on the depositional architecture and internal reflection patterns we identify the wedge to be a contourite drift system^(S1)^. The central and subsidiary mounds in the central GSB are identified as mounded drift units. The moat channel is a result of progressive confinement of currents on the landward side of the mound. Drift formation started shortly after 45 Ma (**Fig. 5a**). Much of the maximum vertical growth (~500 m thickness and ~65 km width) of the central mound was attained during Middle and Late Eocene times. Concomitant to the development of mounded drifts in the central GSB, elongated plastered drifts were formed south of BT (**Fig. 4a**).

The transition between Middle and Late Eocene is marked by a condensed section or a minor hiatus indicated by the absence of the Middle Eocene *Truncarotaloides rohri* zone at the Pukaki-1 well^(S2)^. The presence of *Ismolithus recurves* and *Chiasmolithus oamaruensis* above the hiatus allows assigning an age of ~36 Ma to the upper limit of the hiatus. A similar hiatus is not found at the nearby Pakaha-1 well (**Fig. 2**). The overall current flow path did not change during the entire episode of drift formation. Drift preservation is spatially highly variable. A prominent Middle to Late Oligocene non-depositional or erosional hiatus (Marshall Paraconformity^(S3-S5)^) exists in deeper waters indicating the intensification of currents over a prolonged period that was attributed to the advent of the Antarctic Circumpolar Current (ACC)^(S3)^. While drifts continued to develop during the Oligocene in the central GSB, in the deep-water BT (present water depth >1500 m), the Late Paleocene drift sequence is highly eroded as a result of the vigorous current action that emerged during the Middle to Late Oligocene. We also found remnants of the Middle Eocene drift system on the western bank of the Pukaki Saddle (**Fig. 2**) indicating that the bottom current entered into the GSB through this conduit.

Noteworthy changes in litho- and biostratigraphic records correspond to major changes in basin circulation. Immediately below the basal hiatus of the drift sequence, the Waipawa Formation (25 m thick) is a fine-grained, organic-rich, carbonate-poor mudstone, whose agglutinated benthic foraminifera content indicates a poorly oxygenated environment^(S6)^. Water depths during the Palaeocene were estimated to be 0–100 m^(ref^. ^32)^. A prolonged depositional hiatus (59–49 Ma) at the base of the drift could be due to combined effects of erosion or non-deposition caused by intensified southern-sourced currents. Anomalously rapid subsidence (~1 km) of the Campbell Plateau and the southern Great South Basin took place in the time period between 55 and 45 Ma resulting in a shift from marginal marine to bathyal depths, probably caused by mantle dynamics and lithospheric cooling^(32, 33)^. Following this major event, there was only negligible subsidence throughout the rest of the Paleogene and the Neogene^(32)^. The anomalous subsidence facilitated currents to enter the GSB and BT from the outer margin of the Campbell Plateau. The deep marine sedimentary environment of the GSB shifted to carbonates while clastic sediment supply diminished from the Middle Eocene to the Oligocene^(32)^. Foraminiferal assemblages at the Pukaki-1 well indicate that during the Late Eocene the drift formed at middle and upper bathyal depths under temperate oceanic conditions, while sub-Antarctic conditions prevailed during the Oligocene^(S2)^.

Regional stratigraphic correlation demonstrates that this extensive drift unit predates the sediment drifts that developed since the Early Miocene in the Canterbury Basin (Canterbury drifts, **Fig. 2**)^(S7, 59)^. The Canterbury drift is a consequence of the interaction between the Southland Current and the Bounty Trough cyclonic circulation that originates from the ACC. The development of the drift system prior to the Tasmanian Gateway opening and the resulting establishment of the ACC suggests that the drift system inside the GSB and BT evolved from a southern-sourced Western Boundary Current that was flowing along the outer margin of the Campbell Plateau^(28)^ and that a branch of this current was able to enter into the GSB and BT through the Pukaki Saddle.

## Supplementary Text 2

### Silica and chert as a measure of past diatom productivity

Siliceous microfossils, mainly diatoms, and radiolarians closely reflect surface ocean primary productivity^(S8)^. Diatom productivity is influenced by upwelling of water rich in dissolved silicic acid from greater depths in the open ocean^(53)^. Analysis of past silica productivity events in conjunction with the evaluation of past water mass mixing and tectonic evolution of ocean basins allows the reconstruction of plausible ancient ocean circulation scenarios.

Opaline silica, fixed by siliceous microorganisms, mostly dissolves during its descent through the water column^(S9)^. However, unlike calcium carbonate, opaline silica dissolution does not change with water depth^(S10)^. Preservation of opaline microfossils in marine sediments is strongly dependent on sedimentation rates and diagenesis. Temperature primarily controls the set of reactions that take place during silica diagenesis with minor contributions from the presence or absence of clay, time, pH, pore fluid chemistry, or types of siliceous species^(S11, 24)^. The temperature required for diagenetic conversion varies widely from 2° C to 55° C^(refs.23, 25)^. During diagenesis, amorphous opal-A (original diatoms, radiolarians) dissolves and precipitates as opal-CT (Cristobalite and Tridymite), and subsequently opal-CT recrystallizes to quartz (chert)^(S11-S13)^.

Seismic data calibrated at boreholes are extremely valuable in determining the diagenetic transformation zone regionally and have been used previously to detect silica diagenesis on a regional scale^(S14, S15)^. The opal-A to opal-CT transition is associated with a decrease in porosity, an increase in bulk density, and compressional velocity and can give rise to a positive polarity seismic reflection event that often cross-cuts stratigraphic layers^(23)^. Morphological expressions of the diagenetic front range from simple planar fronts parallel to the host strata (**Fig. S1c**) or cross-cutting positive polarity reflection (**Figs. S1a, S1b and S1d**) to more complex morphologies (saw-tooth patterns, terrace (**Fig. S1c**), and wings (**Fig. S1a**)^(S15, S16)^ and serve as diagnostic criteria of identification in the seismic data. Despite the preservation issues, the diagenetic product, which is preserved as porcellanite or chert, is an excellent indicator of initially deposited biosiliceous ooze in marine sediments and is thus a measure of past biogenic silica productivity.

**Supplementary Text 3**

**Basin-wide silica diagenesis and regional biosiliceous production**

In the seismic lines from the Great South Basin (GSB) a zone with high amplitude reflections stands out clearly between the Late Eocene (36 Ma) and the Early Oligocene and occasionally above the Oligocene strata. In the GSB the top positive polarity reflection bounding the high amplitude zone is mostly parallel to the host strata but in some regions, it cross-cuts local stratigraphy. In the Bounty Trough (BT) and the Canterbury Basin (CB), the high amplitude positive reflection occurs within the latest Eocene through early Miocene strata including a major Middle to Late Oligocene depositional hiatus (Marshall Paraconformity).

At the Pukaki-1 well^(S2)^ in the GSB, the cherty limestone bearing strata correlate with the section of relatively high seismic amplitude and thus serve to calibrate the observations and interpretations of the two-dimensional seismic lines. The Late Eocene is marked by a lithological transition from limestone to overlying cherty limestone (**Fig. 5a**). The base of the chert-bearing sediments occurs within the calcareous nannoplankton zone NP18 (36–36.8 Ma). Chert layers are absent in late Middle Eocene limestone sequence but become more abundant (25–50%) in the Late Eocene and Early Oligocene. The top of the chert-bearing strata is located within the Early Oligocene succession. At this boundary, the sonic log values rapidly decrease downward from an average of 140 μs ft^-1^ to 121 μs ft^-1^ over a short depth range (~20 m) (**Fig. 5a**). This rapid drop is correlated to the transition from soft ooze to underlying chert at the well. Similarly, cherty limestone (36–27 Ma) is underlain by limestone devoid of chert at the Pakaha-1 well^(S17)^, ~55 km west of the Pukaki-1 well. Opal-CT is also present within silicified limestone in the Late Eocene to the Early Oligocene interval at the Integrated Ocean Drilling Program (IODP) Site U1352^(ref^. ^S18)^ located in the CB and provides clear evidence for silica diagenesis. Within the latest Eocene and Oligocene sequence and predominantly in the Oligocene strata, a cross-cutting positive polarity reflector is identified further north of the central GSB. The positive cross-cutting reflection is consistent with an opal-A/opal-CT diagenetic reflection^(23)^. We infer that chert in the limestone bearing strata is a product of silica diagenesis of initially deposited siliceous ooze and the top of the diagenetic transformation boundary is marked by an opal-A to opal-CT reaction front.

We find striking similarities between the inferred silica diagenetic reflector and examples from the northeastern North Atlantic^(S16)^. On a regional scale, an irregular topography of the diagenetic reflector arises from its local relative upward advancement compared to the surrounding region into the Early Miocene strata rich in initially deposited opal. Multi-kilometer scale terraces which are elevated regions of the diagenetic front formed as a result of the irregular upward advancement of the diagenetic boundary (**Fig. S1c**). Terraces are well-developed in the inner Bounty Trough region north of the Bounty channel. Those regions where the diagenetic reaction front moved ahead of the surrounding region are invariably associated with a local collapse of the overburden strata. The collapses are mostly confined to the Miocene succession, and the Pliocene strata are mostly unaffected indicating that opal-A to opal-CT conversion was active during the Late Miocene. In the deeper waters (>1 km) of the BT, the opal-A/opal-CT reflector reveals a distinctly cross-cutting relationship with the host strata. Where it cross-cuts, it appears parallel to the present day seafloor, although in places it appears to be parallel to a Middle Miocene reflector and occasionally to the Late Miocene reflector. The diagenetic front is not in equilibrium with the present day thermal regime where it is not parallel to the seafloor.

Using regional seismic reflection lines, we mapped the opal-A/opal-CT diagenetic reflector in the deeper regions of the GSB, CB and the BT over an area of 25 x 10^4^ km^2^. On a regional scale, the opal-A to opal-CT diagenetic transformation boundary is located approximately 300–1000 m below the seabed (**Fig. 5b**). The present-day temperature at the opal-A to opal-CT conversion boundary is estimated to vary between 23° and 45° C using an average value of the geothermal gradient of 32 °C/km. This temperature range is also consistent with the opal-A to opal-CT phase transition^(23, 25)^. We limited tracing the top of the diagenetic transformation zone (the opal-A/opal-CT diagenetic reflector) to the upper bathyal and shelf regions of the CB and GSB because the late Paleogene siliceous sediments are buried under Neogene prograding sedimentary packages.

At Deep Sea Drilling Project (DSDP) site 277^(ref. S19)^ southwest of the Campbell Plateau (**Fig. 2**) siliceous ooze bearing Late Eocene-Oligocene strata, which are chiefly represented by radiolarian and diatom assemblages, overlie cherty limestone of Early and Middle Eocene age that is devoid of siliceous fossils, most likely due to dissolution during diagenesis. The Early and Middle Eocene cherty limestone contains opal-CT^(S19)^ and provides evidence for dissolution of originally deposited siliceous tests (opal-A) and formation of opal-CT. At this site, the opal-A to opal-CT diagenetic transformation front is located close to the Middle/Late Eocene boundary, ~250 m below the seabed. Presence of diagenetic chert in Middle Eocene strata indicates that the onset of siliceous ooze deposition at site 277 occurred much earlier than at the sites in the north (GSB and BT). In the sedimentary basins north of the Campbell Plateau, such as the GSB and BT, silica production commenced during the latest Eocene and before that calcareous sediments were deposited in the sediment drift system (45–36 Ma). As the deep marine sedimentary record is barren of diagenetic chert, a precursor of initially deposited siliceous tests, it is possible that siliceous microorganisms initially got dissolved in the water column probably because silica was more soluble under initially higher water temperature conditions. However, this possibility is discounted because estimated paleo-water bottom temperatures at DSDP site 277 and the Canterbury Basin were within a close range (10–14° C ^(ref. S20)^) during the Middle Eocene. Site 277 has a prolonged record of siliceous ooze deposition; therefore, silica dissolution in the water column cannot be the cause of the absence of siliceous tests in the sedimentary basins north of Campbell Plateau and east of New Zealand’s South Island during the initial stage of sediment drift evolution. The general absence of chert during 45–36 Ma in these basins thus must reflect the lack of precursor biogenic opal because the water masses were largely impoverished in silicic acid which limited the production of abundant blooms of diatoms.

**Supplementary Text 4**

**Marine connection through the South Tasman Rise prior to and during Tasmanian Gateway opening**

The initial marine connection between the Australo-Antarctic Gulf (AAG) and the southwest Pacific required separation between Antarctica and Tasmania. The South Tasman Rise (STR) is a continental fragment located immediately south of Tasmania. Shearing along the Tasman Fracture Zone on the western margin of the STR started during the earliest Eocene (~53 Ma) and led to initial sinistral strike-slip separation between western STR and Antarctica followed by slow-spreading and fast-spreading (starting at ~43 Ma) until late Eocene (~38 Ma) and subsequent subsidence of the broad shelves in the southern STR marking the demise of the Tasmanian land bridge^(S21)^. The western STR hosts numerous small strike-slip basins. One of them is the Ninene Basin that is up to 135 km wide (Bathymetric image in **Fig. S4**). A shallow marine connection between the AAG and the southwest Pacific could have existed since the Middle to Late Eocene transition through the basins of STR. Accretion of new oceanic crust shortly after ~33.5 Ma^(ref. S22)^ added a deep seaway south of the STR. Results based on Ocean Drilling Program (ODP) Leg 189 drill sites indicate that a deep marine connection through this seaway was possible after ~33.5 Ma^(ref. S21)^ but the direction of current flow is not known. Ref. 16 suggested that the westbound Antarctic Slope Current (ASC) flowed through the Tasmanian Gateway and the eastbound Antarctic Circumpolar Current (ACC) was only enabled after 30 Ma under the influence of the Polar westerlies.

During the development of the continental transform margin (~53 Ma), the western STR experienced both transpressive deformation and minor pull-apart basin formation. The NNW-SSE trending Needwonne Ridge (**Fig. S4**) at the western boundary of the Ninene basin formed as a result of transpression or uplift of a basement block during Eocene strike-slip movements along the Tasman Fracture Zone^(S22)^. By the Late Eocene, the ridge was elevated by 1–2 km from the abyssal plain of the AAG to the west and by ~500 m–1 km compared to coeval strata in the Ninene basin to the east^(S22)^. On the present day bathymetry, the ridge height progressively drops towards the south and terminates in a prominent depression that is ~60 km long and 20 km wide with a rhomb-shaped pull-apart basin to the east. Within the pull-apart basin, a gently convex sedimentary wedge reveals wavy reflectors that prograde upslope and downlap (**Fig. S4**). Based on stratigraphic correlation with the nearest ODP site 1170 in the Ninene Basin, we assign the base of the sedimentary wedge an age of ~38 Ma. Asymmetric wavy sediments were deposited on the hanging wall side to the east (**Fig. S4**). The sedimentary wedge within the depression is interpreted as a contourite. We suggest that the pull-apart basin formed as a result of down-faulting during a phase of continental shearing along the transform margin. The onset of rapid regional tectonic subsidence took place in the middle Late Eocene (36–35 Ma) on the STR (ODP sites 1170 and 1171) and the East Tasman Plateau (Site 1172) as a result of lithospheric stretching that lead to a major subsidence of the continental margins^(48, 21)^. While the Needwonne Ridge could have effectively blocked flow from the AAG into the Ninene basin, the formation of the depression helped to initiate substantial intermediate-depth flow from the AAG to the central STR. The internal gently wavy reflectors (wavelength ~10 km) inside the wedge and asymmetric waves on the hanging wall side of the deposit indicate bottom currents were flowing in a southeast direction. The flow was steered from the AAG into the STR and finally into the Tasman Sea.

Previously, the incursion of westward flowing Antarctic Slope Current carrying the Equatorial Pacific waters was considered to explain the presence of more radiogenic waters at Site 1168 during 33–30 Ma (**Fig. S2b**)^(ref. 16)^. However, while explaining the radiogenic signature, one cannot rule out the possibility of modification of a local water mass circulating at the AAG due to boundary exchange, especially considering the detrital sources. Site 1168 was a shallow restricted basin during Late Eocene and much of Early Oligocene^(S21, S23)^. The terrestrial influence diminished towards the end of Early Oligocene. The site subsided, and pelagic carbonate deposition commenced during Early Oligocene. Since we found evidence of eastbound bottom water flow across the STR since 38 Ma, we don’t favour the argument of westbound waters flowing across the STR towards Site 1168 during 33–30 Ma^(ref. 16)^.

The westerlies likely facilitated easterly-directed shallow (maximum 500 m) flow between 38 and 36 Ma and intermediate-depth flow (~1.5 km) shortly after 35.5 Ma through the marine connection established above the southwestern STR. Another potential route linking the AAG with the South Tasman Sea was the South Tasman Saddle (STS, **Fig. S4**) separating the northern part of the STR from Tasmania. This route likely allowed early flow from the AAG into the Tasman Sea since the Early Eocene^(S24)^. However, the South Tasman Saddle shoaled significantly^(S24)^ due to anomalously high thermal input between 40 and 30 Ma as the active Australia-Antarctic spreading center passed the western end of the South Tasman Saddle, thereby limiting any deep water connection between the AAG and East Tasman Plateau. It may have driven much of the eastbound deep water circulation through the conjugate margins in the western STR. The deepening of the South Tasman Saddle after 30 Ma directly channelled the deep water mass from the AAG into the East Tasman Plateau, which is seen in the change of the Nd isotope signature from equatorial Pacific type water mass to that of the ACC^(16)^.


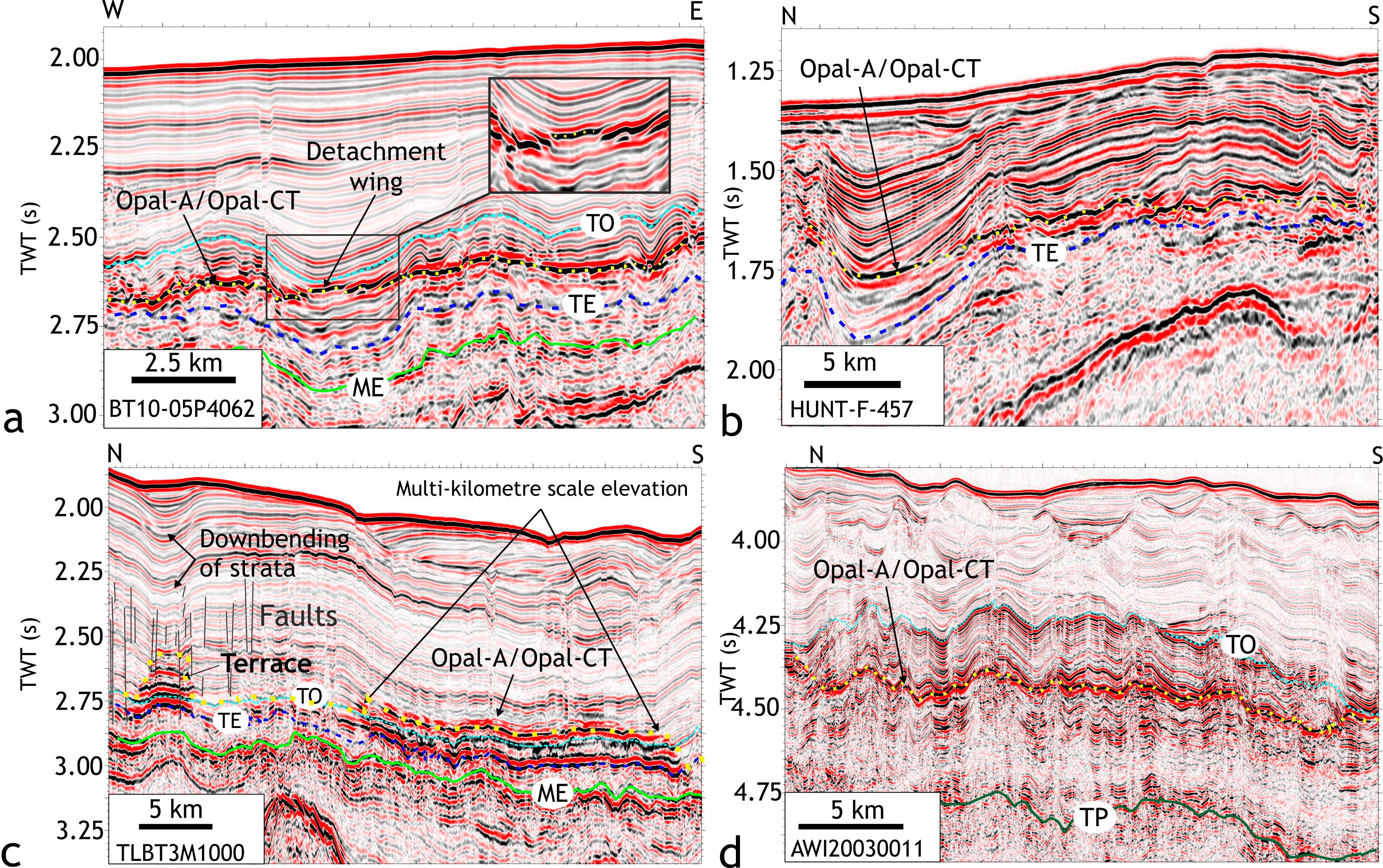


**Fig. S1. Seismic attributes of various morphological features associated with opal-A to opal-CT transformation boundary.** (a) A seismic line showing a strong positive amplitude reflection cross-cutting gently folded Oligocene strata. A minor upward jump of the diagenetic front from the surrounding front is identified as a detachment wing. (b) A cross-cutting opal-A/opal-CT reflector. (c) A seismic line showing terrace (≤2 km) and multi-kilometre scale elevation in which the diagenetic front has preferentially advanced upwards relative to the surrounding regions. Minor faults are present but offsets on those faults are negligible compared to the pronounced downbending of strata above the terrace, and those faults don’t continue to the surface; therefore, downbending is not related to faulting. (d) Reinterpretation of seismic line AWI-20030011^(ref. 34)^ reveals the presence of a cross-cutting, positive amplitude reflector within Late Eocene and Oligocene strata and identified as opal-A to opal-CT transformation boundary. Part of the cross-cutting reflector follows the originally interpreted stratigraphic marker ‘X’^(ref. 34)^, which was assigned an age of Middle Oligocene. Key reflectors are labelled, TO-Top Oligocene, TE-Top Eocene, ME-Middle Eocene, TP=Top Palaeocene. Seismic line locations are shown in **Fig. 5b**.


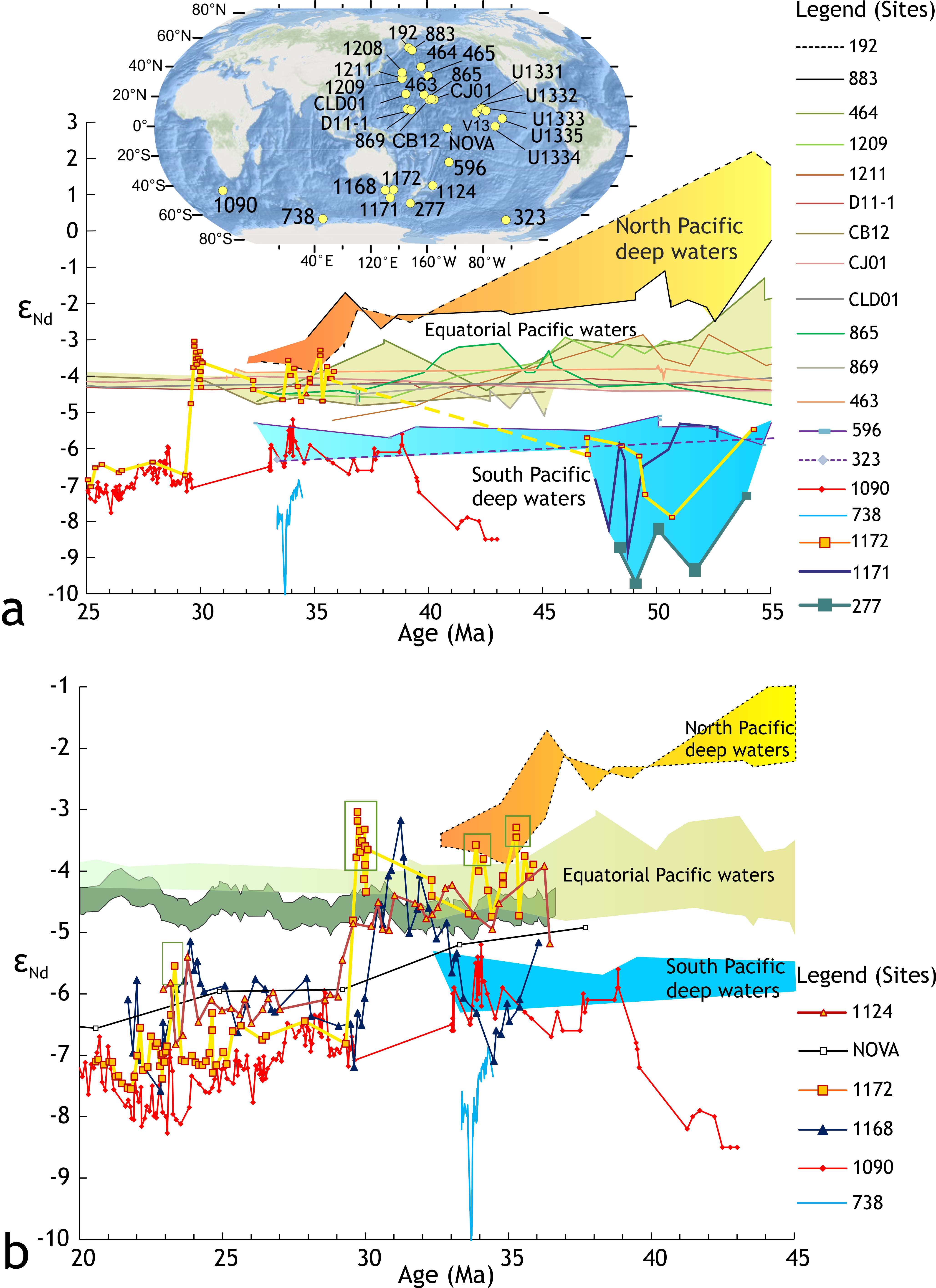


**Fig. S2. Compilation of published ε_Nd_ values (references provided in Supplementary Table S1) from the Pacific Ocean between 55 and 25 Ma.** (a) Paleogene Pacific Ocean Nd isotopic end-member values can be broadly classified into North Pacific deep waters (ε_Nd_(t) = -1–-3) and South Pacific deep waters (ε_Nd_(t) = -5–-9). The ε_Nd_ range of equatorial Pacific Water is intermediate between these two end members. Transient occurrences of extremely negative Nd values (-7 to -10) at Site 277 (52–48 Ma) and at Site 1171(at ~48 and 49 Ma) can be differentiated from the proto-Ross Sea bottom waters (ε_Nd_(t) = -6.0 to -5.0) and interpreted as locally formed intermediate waters in high-latitude South Pacific^(30)^. (b) Comparison of southwest Pacific Nd isotope data from ODP sites 1124, 1172 and 1168 with various Pacific water end members. The dark green ε_Nd_ envelope represents equatorial Pacific Nd isotope data stack from ref. 31. Nd dissolution from volcanic ash in the ODP site 1172 was possible. The main volcanic episode at the East Tasman Plateau was between 40 and 36 Ma^(ref. S25)^. Volcaniclastic sediments are present throughout the late Paleogene and Neogene stratigraphic units in site 1172 but the abrupt disappearance of more radiogenic Nd isotopic values after 29 Ma (such as from -4.8 to -6.8)^(ref. 16)^ is not due to the absence of volcanic inputs rather a switch from equatorial pacific deep waters to the ACC. The peak radiogenic Nd isotopic signatures (ε_Nd_(t)>-4) marked by green rectangles may have originated from a partial dissolution of intermittent transient volcanic inputs.


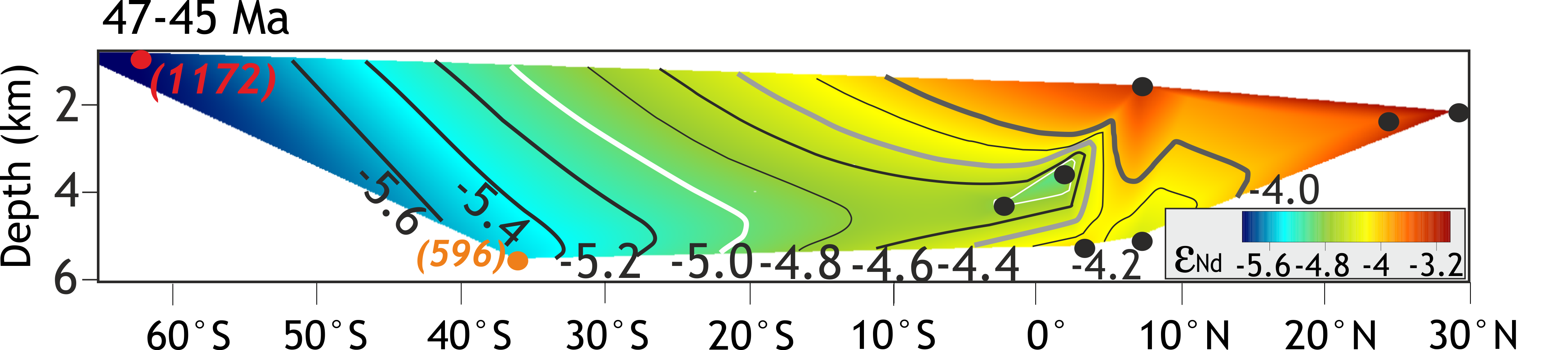


**Fig. S3.** Neodymium isotopic section from the Pacific Ocean for 47 and 45 Ma time bin interpolated using natural neighbor interpolation (**Table S1**).


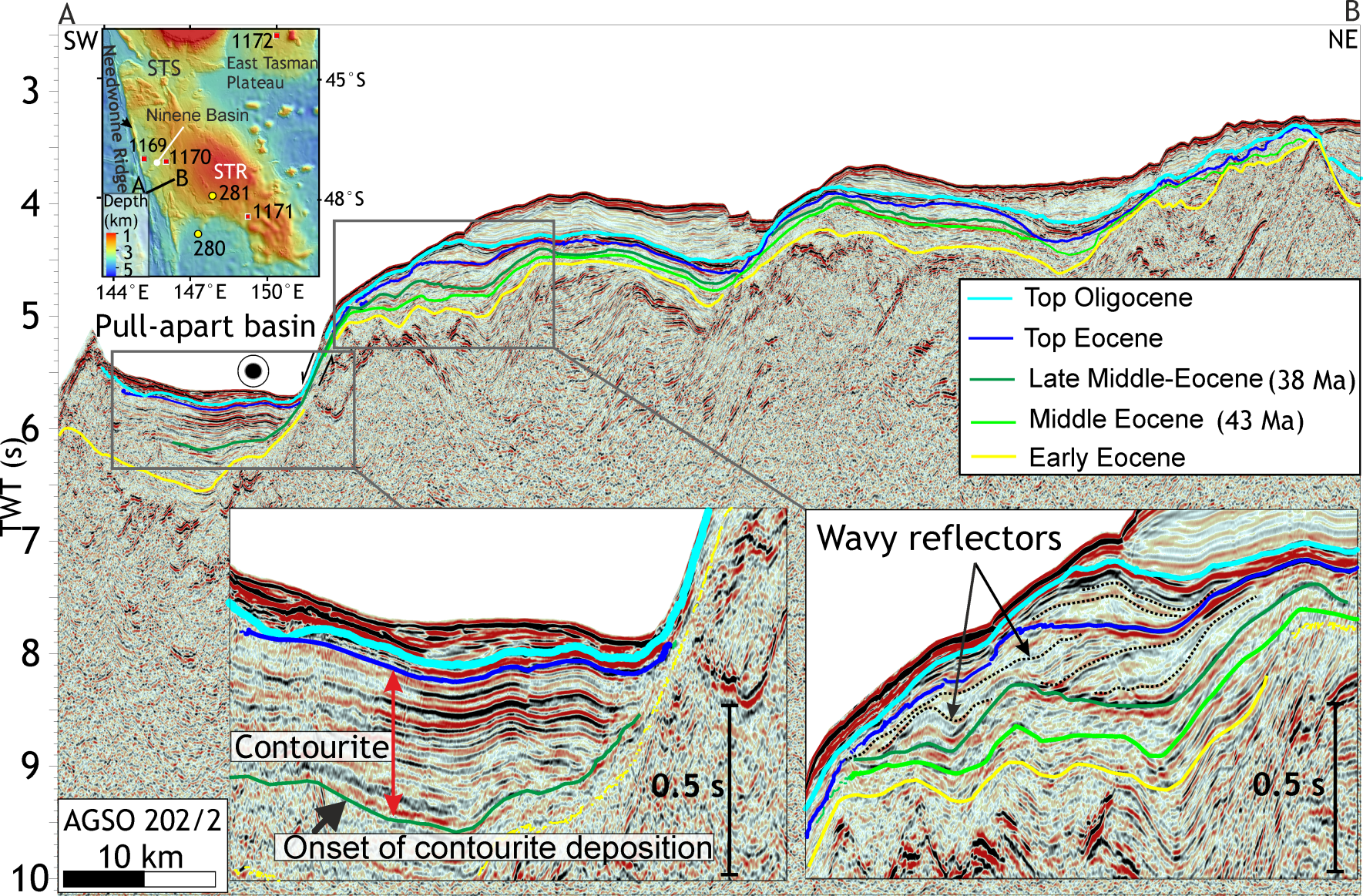


**Fig. S4.** **Map and seismic geometry of contouritic drifts in the South Tasman Rise (STR).** Locations of scientific drill holes are shown on the present-day bathymetric image (GEBCO_08 grid^(ref. S26)^). The Needwonne Ridge at the western margin of the STR is a transpressive structure formed due to shearing along the Tasman Fracture Zone. The South Tasman Saddle (STS) separates the STR from Tasmania. A seismic line (AB) shows a sedimentary drift deposited by a southeasterly flowing bottom-current (considering the paleogeographic position of the South Tasman Rise at 35 Ma as shown in Fig. 6) within a depression. Presence of a contouritic drift indicates the role of initial weak bottom-currents. Insets in the bottom show upslope migrating and downlapping reflectors in the depression and asymmetric waves on the adjacent slope to the northeast.

**Table S1**. **Compilation of published Pacific Nd isotope results.** Paleo-locations are obtained from GPlates. Paleo-depths for sites 1168 and 1172 were obtained from ref. 16. Paleo-depths for Ferromanganese crust sites and site 1124 were obtained from the paleo-bathymetry grids generated by the Earthbyte group^(S27)^. Paleo-depths for the rest of the sites were compiled from ref. 26.

**a. Compilation for 45–47 Ma**

| **Site** | **Palaeodepth (km)** | **Palaeo-latitude** | **ε_Nd_(t)** | **Reference** | **Sample type** |
| --- | --- | --- | --- | --- | --- |
| 464 | 2.1 | 29 | -3 | Hague et al., 2012 (ref. 69) | Fossil fish debris |
| 1209 | 2.3 | 24.3 | -3.57 | Thomas, 2004 (ref. 68) | Fossil fish debris |
| D11-1 | 5.2 | 3.18 | -4.07 | Ling et al., 1997 (ref. 67) | Ferromanganese crust |
| V13-2 | 4.25 | -2.35 | -4.84 | Ling et al., 1997 (ref. 67) | Ferromanganese crust |
| CB12 | 5.00 | 7.14 | -4.43 | Ling et al., 2005 (ref. 70) | Ferromanganese crust |
| 865 | 1.5 | 7.3 | -3.3 | Thomas, 2004 (ref. 68) | Fossil fish debris |
| 869 | 3.5 | 1.75 | -5.1 | Thomas et al., 2014 (ref. 26) | Fossil fish  debris |
| 596 | 5.5 | -36.16 | -5.5 | Thomas et al., 2014 (ref. 26) | Fossil fish  debris |
| 1172 | 0.75 | -62.5 | -6.2 | Huck et al., 2017 (ref. 30) | Fossil fish  debris |

**b. Compilation for 36.5–38 Ma**

| **Site** | **Palaeodepth (km)** | **Palaeo-latitude** | **ε_Nd_(t)** | **Reference** | **Sample type** |
| --- | --- | --- | --- | --- | --- |
| 192 | 3 | 43.5°N | -2.5 | Hague et al., 2012 (ref. 69) | Fossil fish debris |
| 464 | 4.5 | 29.75°N | -3.1 | Hague et al., 2012 (ref. 69) | Fossil fish debris |
| 883 | 2.3 | 40.94°N | -2.3 | Hague et al., 2012 (ref. 69) | Fossil fish debris |
| 884 | 3.7 | 41.79°N | -3.7 | Hague et al., 2012 (ref. 69) | Fossil fish debris |
| 1209 | 2.3 | 24.06°N | -4.6 | Thomas, 2004 (ref. 68) | Fossil fish debris |
| 1211 | 2.9 | 23.56°N | -4.81 | Thomas, 2004 (ref. 68) | Fossil fish debris |
| CD29-2 | 2 | 6°N | -4.67 | Ling et al., 1997 (ref. 67) | Ferromanganese crust |
| D11-1 | 1.8 | 3.65°N | -4.25 | Ling et al., 1997 (ref. 67) | Ferromanganese crust |
| VA13-2 | 4 | 1.36°S | -4.72 | Ling et al., 1997 (ref. 67) | Ferromanganese crust |
| CB12 | 2 | 8°N | -4.81 | Ling et al., 2005 (ref. 70) | Ferromanganese crust |
| CJ01 | 3 | 8°N | -4.12 | Ling et al., 1997 (ref. 67) | Ferromanganese crust |
| CLD01 | 2 | 13.8°N | -4.2 | Ling et al., 2005 (ref. 70) | Ferromanganese crust |
| NOVA(D137-09) | 6.5 | 11°S | -4.92 | van de Flierdt et al., 2004 (ref. S28) | Ferromanganese crust |
| 865 | 1.5 | 7.79°N | -4.5 | Thomas et al., 2014 (ref. 26) | Fossil fish debris |
| 869 | 3.5 | 2.08°N | -4.5 | Thomas et al., 2014 (ref. 26*)* | Fossil fish debris |
| 596 | 5.5 | 35.26°S | -5.7 | Thomas et al., 2014 (ref. 26) | Fossil fish debris |
| 323 | 5 | 66.15°S | -6.3 | Thomas et al., 2014 (ref. 26) | Fossil fish debris |
| 1124 | 2.5 | 49°S | -5.13 | Scher et al., 2015 (ref.16) | Fossil fish debris |

**c. Compilation for 36–34 Ma**

| **Site** | **Palaeodepth (km)** | **Palaeo-latitude** | **ε_Nd_(t)** | **Reference** | **Sample type** |
| --- | --- | --- | --- | --- | --- |
| 192 | 3 | 43.5°N | -3.5 | Hague et al., 2012 (ref. 69) | Fossil fish debris |
| 464 | 4.5 | 29.75°N | -3.8 | Hague et al., 2012 (ref. 69) | Fossil fish debris |
| 883 | 2.3 | 40.94°N | -1.7 | Hague et al., 2012 (ref. 69) | Fossil fish debris |
| 884 | 3.7 | 41.79°N | -1.7 | Hague et al., 2012 (ref. 69) | Fossil fish debris |
| 1209 | 2.3 | 24.06°N | -4.6 | Thomas, 2004 (ref. 68) | Fossil fish debris |
| 1211 | 2.9 | 23.56°N | -5.22 | Thomas, 2004 (ref. 68) | Fossil fish debris |
| CD29-2 | 2 | 7.7°N | -4.11 | Ling et al., 1997 (ref. 67) | Ferromanganese crust |
| D11-1 | 1.8 | 4.5°N | -4.2 | Ling et al., 1997 (ref. 67) | Ferromanganese crust |
| VA13-2 | 4 | 0° | -4.59 | Ling et al., 1997 (ref. 67) | Ferromanganese crust |
| CB12 | 2 | 9.8°N | -4.7 | Ling et al., 2005 (ref. 70) | Ferromanganese crust |
| CJ01 | 3 | 9.5°N | -4.1 | Ling et al., 1997 (ref. 67) | Ferromanganese crust |
| CLD01 | 2 | 15°N | -4.2 | Ling et al., 2005 (ref. 70) | Ferromanganese crust |
| NOVA | 6.5 | 10°S | -5 | van de Flierdt et al., 2004 (ref. S28) | Ferromanganese crust |
| 865 | 1.5 | 7.79°N | -4.4 | Thomas et al., 2014 (ref. 26) | Fossil fish debris |
| 869 | 3.5 | 2.08°N | -4.2 | Thomas et al., 2014 (ref. 26) | Fossil fish debris |
| 596 | 5.5 | 35.26°S | -5.6 | Thomas et al., 2014 (ref. 26) | Fossil fish debris |
| 323 | 5 | 66.15°S | -6.3 | Thomas et al., 2014 (ref. 26) | Fossil fish debris |
| 1124 | 3 | 48°S | -4.00 | Scher et al., 2015 (ref.16) | Fossil fish debris |
| 1168 | 0.25 | 61°S | -6.16 | Scher et al., 2015 (ref. 16) | Fossil fish debris |
| 1172 | 2.25 | 61°S | -4.2 | Scher et al., 2015 (ref. 16) | Fossil fish debris |

**d. Compilation for 34–33 Ma**

| **Site** | **Palaeodepth (km)** | **Palaeolatitude** | **ε_Nd_(t)** | **Reference** | **Sample type** |
| --- | --- | --- | --- | --- | --- |
| 192 | 3 | 43.5°N | -3.7 | Hague et al., 2012 (ref. 69) | Fossil fish debris |
| 464 | 4.5 | 29.75°N | -4.4 | Hague et al., 2012 (ref. 69) | Fossil fish debris |
| 883 | 2.3 | 40.94°N | -2.9 | Hague et al., 2012 (ref. 69) | Fossil fish debris |
| 884 | 3.7 | 41.79°N | -1.8 | Hague et al., 2012 (ref. 69) | Fossil fish debris |
| 1209 | 2.3 | 24.06°N | -4.5 | Thomas, 2004 (ref. 68) | Fossil fish debris |
| CD29-2 | 2 | 8°N | -4 | Ling et al., 1997 (ref. 67) | Ferromanganese crust |
| D11-1 | 1.8 | 5°N | -4.3 | Ling et al., 1997 (ref. 67) | Ferromanganese crust |
| VA13-2 | 4 | 0° | -4.8 | Ling et al., 1997 (ref. 67) | Ferromanganese crust |
| CB12 | 2 | 10°N | -4.6 | Ling et al., 2005 (ref. 70) | Ferromanganese crust |
| CJ01 | 3 | 10°N | -4.0 | Ling et al., 1997 (ref. 67) | Ferromanganese crust |
| CLD01 | 2 | 15°N | -4.2 | Ling et al., 2005 (ref. 70) | Ferromanganese crust |
| NOVA | 6.5 | 9°S | -5.2 | van de Flierdt et al., 2004 (ref. S28) | Ferromanganese crust |
| 865 | 1.5 | 7.79°N | -4.5 | Thomas et al., 2014 (ref. 26) | Fossil fish debris |
| 596 | 5.5 | 35.26°S | -5.4 | Thomas et al., 2014 (ref. 26) | Fossil fish debris |
| 323 | 5 | 66.15°S | -6.3 | Thomas et al., 2014 (ref. 26) | Fossil fish debris |
| 1124 | 3 | 47°S | -4.2 | Scher et al., 2015 (ref. 16) | Fossil fish debris |
| 1168 | 0.48 | 60°S | -5.7 | Scher et al., 2015 (ref. 16) | Fossil fish debris |
| 1172 | 2.5 | 60°S | -4.0 | Scher et al., 2015 (ref. 16) | Fossil fish debris |

**Age model:** Ages were determined for sites 1209 and 1211 by linearly interpolating sedimentation rates between biostratigraphic datums determined from the results of ODP Leg 198^(refs. S29, 68)^. Calcareous nannofossil datums reported in ref. S29 were assigned numeric ages from the Geologic Time Scale 2004^(ref. S30)^. Ferromanganese crust ages were determined by determining the growth rates of the crusts from combined Co constant flux calculations and ^10^Be/^9^Be ratios^(refs. 67, 70, S28)^. The age models for DSDP Site 596 and ODP Sites 865 and 869 were updated to Geologic Time Scale 2004^(ref. 26)^. A constant Co accumulation age model was used to assign numeric ages to the sediment intervals at the DSDP Site 596^(ref. 26)^. Nannofossil datums for ODP Sites 865 and 869 were assigned numeric ages from ref. S30. Biostratigraphic datums and average sedimentation rates were used to define the age model at the DSDP Site 323 (ref. 26). At this site it was not possible to convert the shipboard age estimates to the Geologic Time Scale 2004; therefore, the ages at this site can be treated as broad estimates. Age models for ODP sites 1124, 1172, and 1168 are from refs. S31, S32, S33. The age model is based on using both paleomagnetic and biostratigraphic data from the ODP Leg 181^(ref. S34)^ and the ODP Leg 189 initial results^(ref. S21)^ and the timescale of ref. S35 was used.

**Supplementary references not in the main manuscript**

S1. M. Rebesco, F. J. Hernandez-Molina, D. Van Rooij, A.Wahlin, Contourites and associated sediments controlled by deep-water circulation processes: State-of-the-art and future considerations. *Marine Geology* **352**, 111–154 (2014). doi:10.1016/j.margeo.2014.03.011

S2. Placid Oil Company. "Final Geological Well Report Pukaki-1 Great South Basin New Zealand PPL 38081", Ministry of Economic Development New Zealand open-file Petroleum Report PR1005 (1984)

S3. R. M. Carter, C. A. Landis, Correlative Oligocene Unconformities in Southern Australasia. Nature Physical Science 237, 12–13 (1972). doi:10.1038/physci237012a0

S4. D. G. Jenkins, Initiation of the proto circum-Antarctic current. Nature 252, 371–373 (1974). doi:10.1038/252371a0

S5. C. S. Fulthorpe, R. M. Carter, K. G. Miller, J. Wilson, Marshall Paraconformity: A mid-Oligocene record of inception of the Antarctic Circumpolar Current and coeval glacio-eustatic lowstand? Marine and Petroleum Geology 13, 61–77 (1996). doi:10.1016/0264-8172(95)00033-X

S6. P. Schiøler et al., Palynofacies, organic geochemistry and depositional environment of the Tartan Formation (Late Paleocene), a potential source rock in the Great South Basin, New Zealand. *Marine and Petroleum Geology* **27**, 351–369 (2010). doi:10.1016/j.marpetgeo.2009.09.006

S7. C. S. Fulthorpe, R. M. Carter, Continental-shelf progradation by sediment-drift accretion. *Geological Society of America Bulletin* **103**, 300–309 (1991). doi:10.1130/0016-7606(1991)103<0300:CSPBSD>2.3.CO;2

S8. A. P. Lisitzin, Distribution of siliceous microfossils in suspension and in bottom sediments, in The micropaleontology of the oceans, M. N. Funnell, W. R. Riedel, Eds. (Cambridge University Press, Cambridge, England, C1971) p. 173-195.

S9. R. Wollast, The silica problem, in *The Sea: Marine Chemistry* (5), E. D. Goldberg, Ed. (Wiley, New York, 1974), pp. 359–392.

S10. G. R. Heath, Dissolved Silica and Deep-Sea Sediments, in *Studies in Paleo-Oceanography* (SP20), W. W. Hay, Ed. (The Society of Economic Paleontologists and Mineralogists, Tulsa, 1974), pp. 77–93.

S11. M. Kastner, J. B. Keene, J. M. Gieskes, Diagenesis of Siliceous Oozes.1. Chemical Controls on Rate of Opal-A to Opal-CT Transformation - Experimental-Study. *Geochimica Et Cosmochimica Acta* **41**, 1041–1059 (1977). doi:10.1016/0016-7037(77)90099-0

S12. C. M. Isaacs, Porosity Reduction During Diagenesis of Monterey Formation, Santa Barbara Coastal Area, California, in *The Monterey Formation and related Siliceous Rocks of California*, R. E. Garrison, R. G. Douglas, Eds. (Society of Economic Paleontologists and Mineralogists, San Francisco, 1981), pp. 257–271.

S13. R. Tada, Compaction and cementation in siliceous rocks and their possible effect on bedding enhancement, in *Cycles and Events in Stratigraphy*, G. Einsele Ed. (Springer Verlag, Berlin-Heidelberg, 1991), pp. 480–491.

S14. R. J. Davies, J. Cartwright, A fossilized Opal A to Opal C/T transformation on the northeast Atlantic margin: support for a significantly elevated Palaeogeothermal gradient during the Neogene? *Basin Research* **14**, 467–486 (2002). doi:10.1046/j.1365-2117.2002.00184.x

S15. D. Meadows, R. J. Davies, Morphological development of basin-scale silica diagenetic fronts revealed with 2D seismic reflection data: offshore Sakhalin, Russian Far East. *Journal of the Geological Society* **164**, 1193–1206 (2007). doi:10.1144/0016-76492006-163

S16. R. J. Davies, Differential compaction and subsidence in sedimentary basins due to silica diagenesis: A case study. *Geological Society of America Bulletin* **117**, 1146–1155 (2005). doi:10.1130/b25769.1

S17. HIPCO, Pakaha-1 Well Completion Report, Ministry of Economic Development New Zealand open-file Petroleum Report PR703 (1977).

S18. Expedition 317 Scientists, Site U1352, in *Proc. IODP, 317*, C. S. Fulthorpe, K. Hoyanagi, P. Blum, and the Expedition 317 Scientists, (Integrated Ocean Drilling Program Management International, Inc., Washington, D. C., 2011). pp. 1–171. doi:10.2204/iodp.proc.317.104.2011

S19. Shipboard Scientific Party, Site 277, in *Initial Reports of the Deep Sea Drilling Project*, Volume 29, S. M. White, Ed. (U.S. Government Printing Office, Washington, 1975), pp. 45–120. doi:10.2973/dsdp.proc.29.104.1975

S20. C. J. Hollis et al., Early Paleogene temperature history of the Southwest Pacific Ocean: Reconciling proxies and models. *Earth and Planetary Science Letters* **349**, 53–66 (2012). doi: 10.1016/j.epsl.2012.06.024

S21. Shipboard Scientific Party, Leg 189 Summary, in *Proceedings of ODP Initial Reports* (189) in N. F. Exon, J. P. Kennett, M. J. Malone, Eds. (Ocean Drilling Program, College Station, Texas, 2001), pp. 1–98.

S22. N. F. Exon, A. M. G. Moore, P. J. Hill, Geological framework of the South Tasman Rise, south of Tasmania, and its sedimentary basins, *Australian Journal of Earth Sciences*, **44**:5, 561–577(1997), doi: 10.1080/08120099708728337.

S23. Shipboard Scientific Party, Site 1168, in Proceedings of ODP Initial Reports (189) in N. F. Exon, J. P. Kennett, M. J. Malone, Eds. (Ocean Drilling Program, College Station, Texas, 2001), doi:10.2973/odp.proc.ir.189.103.2001.

S24. L. A. Lawver, L. M. Gahagan, I. W. D. Dalziel, A Different Look at Gateways: Drake Passage and Australia/Antarctica, in *Tectonic, Climatic, and Cryospheric Evolution of the Antarctic Peninsula*, J. B. Anderson, J. S. Wellner, Eds. (American Geophysical Union, Washington, D. C. , 2011). doi: 10.1029/2010SP001017.

S25. N. F. Exon *et al.*, Geological evolution of the East Tasman Plateau, a continental fragment southeast of Tasmania. *Australian Journal of Earth Sciences* **44**:5, 597-608 (1997). doi:10.1080/08120099708728339

S26. W. H. F. Smith, D. T. Sandwell, Global sea floor topography from satellite altimetry and ship depth soundings. *Science* **277**, 1956-1962 (1997). doi:10.1126/science.277.5334.1956

S27. R. D. Müller, M. Sdrolias, C. Gaina, B. Steinberger, C. Heine, Long-term sea-level fluctuations driven by ocean basin dynamics. Science **319**, 1357–1362 (2008).

10.1126/science.1151540.

S28. T. van de Flierdt, M. Frank, J. R. Hein, B. Hattendorf, D. Gunther, P. W. Kubik, Deep and bottom water export from the Southern Ocean to the Pacific over the past 38 million years, *Paleoceanography*, v. **19**, PA1020 (2004).doi:10.1029/2003PA000923.

S29. T. J. Bralower *et al.*, Proc. ODP Init. Rep. [online] 198, (http://www- odp.tamu.edu/publications/198_IR/chap_05/chap_05.htm) (http://www-odp.tamu.edu/publications/198_IR/chap_07/chap_07.htm) (2002).

S30. F. M. Gradstein, J. G. Ogg, A. B. Smith, A Geological Time Scale 2004. (Cambridge Univ. Press, Cambridge, U. K., 2004), 610 pp.

S31. L. H. Joseph, D. K. Rea, B. A. van der Pluijm, Neogene history of the Deep Western Boundary Current at Rekohu sediment drift, Southwest Pacific (ODP Site 1124). *Mar. Geol.* 205, 185–206 (2004).

S32. C. E. Stickley et al., Late Cretaceous to Quaternary biomagnetostratigraphy of ODP Sites 1168, 1170, 1171, and 1172, Tasmanian Gateway. Proc. ODP Sci. Res http://www-odp.tamu.edu/publications/189_SR/111/111.htm (2004).

S33. M. Fuller, Y. Touchard, On the Magnetostratigraphy of the East Tasman Plateau Timing of the Opening of the Tasmanian Gateway and Paleoenvironmental Changes, in *The Cenozoic Southern Ocean. Tectonics, Sedimentation, and Climate Change between Australia and Antarctica*, N. F. Exon, J. P. Kennett, M. Malone, Eds. (American Geophysical Union, 2004), pp. 63–78.

S34. R. M. Carter *et al.*, Proceedings of the Ocean Drilling Program, Initial Results, 181, 80. (1999).

S35. W. A. Berggren, D.V. Kent, C. C. Swisher III, M.-P. Aubry, A revised Cenozoic geochronology and chronostratigraphy, in *Geochronology, Time Scales and Global Stratigraphic Correlation,* W. A. Berggren, D.V. Kent, M. P. Aubry, and J. Hardenbol, Eds. (Spec. Publ.Soc. Econ. Paleontol. Mineral., Soc. Sediment. Geol., 1995), 54:129–212.
